# The Application Value of Magnetic Resonance Imaging (MRI) in the Clinical Diagnosis in Hospital Management

**DOI:** 10.12669/pjms.37.6-WIT.4791

**Published:** 2021

**Authors:** Yan Chen, Hong-Mei Zhou, Qi-Jie Jiang

**Affiliations:** 1 Yan Chen, Attending Doctor Business School, Sichuan University, Chengdu, China; 2 Hong-Mei Zhou, Attending Doctor, School of Public Administration, Sichuan University, Chengdu, China; 3 Qi-Jie Jiang, Associate Professor Business School, Chengdu University, Chengdu, China

**Keywords:** Magnetic resonance imaging, Fuzzy C-means clustering algorithm, Curative effect, Clinical diagnosis, Hospital management

## Abstract

**Background & Objective:**

To evaluate the application value of dynamic enhanced magnetic resonance imaging (DCE-MRI) in the clinical diagnosis in hospital based on the fuzzy C-means clustering algorithm and conduct its cost-benefit analysis in practice.

**Methods:**

This study was conducted from October 11th 2020 to November 25, 2020. Eighteen patients with lung cancer confirmed by pathology were collected and examined by DCE-MRI one day before and one month after surgery using a fuzzy C-means clustering algorithm. And then, taking Guang’an District People’s Hospital as an example, the present paper conducts the cost-benefit analysis with the utilization of net present value (NPV).

**Results:**

After 1-month review, eleven patients with effective treatment, four numerical Ktrans, Kep, Ve, Vp compared with preoperative decreased, which Ktrans, Kep, the change of the Ve was statistically significant (P < 0.05), the Vp of decline has no statistical significance (P > 0.05), the experimental and ineffective group after an operation, compared to four numerical Ktrans and Vp difference was statistically significant (P < 0.05), the corresponding AUC (area under the curve) to 1 and 0.818, the sensitivity of 100% and 85.7%, a specific degree of 100% and 72.7%. The NPV analysis results showed that the hospital could achieve profit and loss balance in the third year after purchasing MRI equipment and then gradually obtain positive cash inflow.

**Conclusion:**

The application of MRI in the Clinical Diagnosis helps hospital promote the therapeutic effect, thus having great practical value, and also contributes to increasing benefits in the long run.

**Abbreviations:**
DCE-MRI: Dynamic enhanced magnetic resonance imaging.
NPV: Net Present Value.
RECIST: Reliable Tumor Evaluation Criteria.
CR: Complete Response.

## INTRODUCTION

It is of great significance to carry out quality management on the laboratory and improve the quality level of the laboratory. Dynamic enhanced magnetic resonance imaging (DCE-MRI) is an emerging functional imaging technology in recent years.^1^-^3^ A series of parameters reflecting the hemodynamic changes of tumor micro-vessels are obtained through intravenous contrast agent injection and continuous multi-phase dynamic scanning.^4^ For example, at present, the incidence of lung cancer in China remains high. According to statistics, male lung cancer patients account for the first cause of cancer death, second only to breast cancer among women. Interventional therapy can be an effective treatment for patients who cannot accept surgery or are intolerant to chemo radiotherapy. However, some realistic problems need to be considered in the practice: can the hospital make a profit after purchasing the MRI equipment because the cost of MRI equipment is usually high? How long can it be profitable? This paper attempts to focus on these questions.

## METHODS

Firstly, we used a 3.0T superconducting MAGNETIC resonance scanner (Discovery 750), eight-channel phased array coil, plus respiratory gating. All patients were subjected to a general MRI scan: axial T2PROPELLER (TR6300ms, TE75ms, layer thickness 5mm, layer spacing 1mm, FOV40cm), Scanning time: 2min15s; DWI (B =800s/mm2, TR5700ms, TE50ms, layer thickness 5mm, layer spacing 2mm, FOV40cm), Scan time 50S. Then scanned for DCE-MRI: first sweep 5 turn Angle of the Flip angle 3, 6, 9, 12, 15, scanning sequence for BHAxLAVA - Flex - 3, 6, 9, 12, 15, scanning parameters for TR3.6 ms, TE1.7 ms, 5 mm, with a thick layer of layer 1 mm spacing, FOV40cm, each sequence scan time is 6s, then dynamic contrast enhancement scan sequence BHAxLAVA - Flex - 12 + C, scanning parameters for TR3.6 ms, TE1.7 ms, 5 mm, with a thick layer of layer spacing of 1 mm, FOV40cm, before injection of contrast agent, the first phase was straightforward scanning, followed by high pressure injection of gadolinium diamine contrast agent (0.1mmol/kg) at an injection rate of 2ml/s, and injection of 0.9% normal saline at the same rate of 20ml, a total of 50 scanning periods, 6s per phase, for a total of 5min. All patients were given routine therapy after surgery^5^ and reliable tumor evaluation criteria (RECIST) were used to evaluate the efficacy.

Secondly, we got five turn Angle and dynamic contrast-enhanced image import OK software. ROI was delineated at the same level for each patient before and after surgery, avoiding necrotic cystic changes and normal blood vessels, and four quantitative parameters were calculated: Ktrans (volume transfer constant), Kep(interstitial - plasma rate constant), Ve(extracellular space volume fraction), and Vp(plasma volume fraction). Finally, based on the annual cash flow of MRI, the cost of MRI is analyzed by calculating the net present value.^6^

## RESULTS

After one month of treatment, the DCE-MRI images of 11 patients showed that the substantial part of the tumor’s diameter decreased by more than 30%, indicating that the treatment was effective. The seven patients belonged to SD or PD, that is, the invalid group and the change of the red area on the false-color map was not noticeable, or the range was more extensive than before. In the influential group, Ktrans, Kep, Ve, and Vp were all decreased compared with those before treatment, and the changes in Ktrans, Kep, and Ve were statistically significant (P=0.002, P=0.01, P=0.036), while the difference in Vp was not statistically significant (P=0.084). Postoperative Ktrans and Vp of the influential group and the non-effective group showed statistically significant differences (P=0.001, P=0.036), corresponding AUC (area under the curve) were 1 and 0.818, sensitivity 100% and 85.7%, specificity 100%, and 72.7%, as shown in Table-I-III. Relevant ROC curves of Ktrans, Kep, Ve, and Vp in all patients after surgery.

**Table-I:** Paired sample T-test of preoperative and postoperative parameters in the effective group.

*Time*	*Ktrans/(min-1)*	*Kep/(min-1)*	*Ve*	*Vp*
preoperative	0.321±0.235	0.828±0.385	0.205±0.108	0.085±0.098
postoperative	0.044±0.028	0.348±0.155	0.111±0.062	0.079±0.108
P	0.002	0.01	0.036	0.084

**Table-II:** Postoperative independent sample T-test of the effective group and the invalid group.

*Grouping*	*Ktrans/(min-1)*	*Kep/(min-1)*	*Ve*	*Vp*
Effective group	0.044±0.028	0.348±0.155	0.111±0.062	0.079±0.108
The invalid group	0.397±0.285	0.519±0.323	0.186±0.11	0.311±0.312
P	0.001	0.148	0.081	0.036

**Table-III:** AUC, maximum approximate index, sensitivity, and specificity of the influential group and the non-effective group after operation.

*Statistical indicators*	*Ktrans*	*Kep*	*Ve*	*Vp*
AUC	1	0.727	0.727	0.818
Maximum approximation index	1	0.584	0.494	0.584
The sensitivity	1	0.857	0.857	0.857
Specific degrees	1	0.727	0.636	0.727

And then, taking Guang’ an District People’s Hospital as an exam ple, the present paper conducts the cost-benefit analysis with the utilization of net present value (NPV). The annual net present value of MRI is about two million RMB. The total investment income of MRI in 2001 and 2002 reached 75% of the investment cost of the equipment. In the third year, positive cash inflow can be realized. Through the analysis of 5-year complete data of MRI in hospital by year, through the study of cost benefit development trend, it shows that with the operation of MRI, its economic benefits increase year by year. The NPV analysis results showed that the hospital could achieve profit and loss balance in the third year after purchasing MRI equipment and then gradually obtain positive cash inflow.

## DISCUSSION

According to the two-compartment hemodynamic model proposed in the literature,^7^ tumor microcirculation characteristics, such as vascular size, distribution, permeability,^8^-^10^ etc. were evaluated using quantitative methods. Four parameters, Ktrans, Kep, Ve, and Vp, were obtained by fitting calculation.

The difference in Ktrans and Vp changes in the effective group and the ineffective group after treatment was significant. Among the four values, Ktrans had the highest sensitivity and specificity, indicating that Ktrans was used to evaluate tumor blood perfusion and predict the therapeutic effect, which was consistent with most scholars.^11^,^12^

The advantages of using dynamic contrastive enhanced MAGNETIC resonance quantitative parameters to evaluate the efficacy of lung cancer are as follows: first, morphological changes of the lesions and the supply of tumor microvessels can be observed at the same time to obtain rich diagnostic information, and the evaluation method is more accurate and objective.13 Second, this examination has no radiation effect and is more suitable for long-term follow-up reviews than CT and other examination methods. Third, the examination cost is moderate, easy to be accepted by patients.^14^ These results are consistence with several previous studies, for example, Zheng et al.^15^

## CONCLUSIONS

DCE-MRI can effectively evaluate the curative effect of BACE treatment in lung cancer patients, which is objective, accurate, and non-invasive, and provides a new reference basis for clinical adjustment of the treatment plan. However, large sample data support and long-term follow-up in-depth study are still needed. And the NPV analysis results showed that the hospital could achieve profit and loss balance in the third year after purchasing MRI equipment and then gradually obtain positive cash inflow and the net cash inflow in each year is increasing continuously.

### Authors Contribution:

**YC:** Conceived, designed and did statistical analysis & editing of manuscript.

**HMZ:** Did data collection and manuscript writing.

**QJJ:** Takes the responsibility and is accountable for all aspects of the work in ensuring that questions related to the accuracy or integrity of any part of the work are appropriately investigated and resolved.
